# Evaluation médico-légale des certificats médicaux initiaux au sein des structures sanitaires du Nord de la Tunisie

**DOI:** 10.11604/pamj.2021.40.255.28573

**Published:** 2021-12-22

**Authors:** Chahnez Makni, Myriam Gorgi, Meriem Gharbaoui, Sarra Ben Abderrahim, Mohamed Amine Zaara, Azza Belhaj, Moncef Hamdoun, Mohamed Allouche

**Affiliations:** 1 Service de Médecine Légale, Hôpital Charles-Nicolle, 138, Boulevard 9-Avril-1938, 1006 Tunis, Tunis, Tunisie,; 2 Faculté de Médecine de Tunis, Université Tunis El Manar, Tunis, Tunisie

**Keywords:** Certificat médical initial, traumatisme, évaluation, Initial medical certificate, trauma, assessment

## Abstract

Le certificat médical initial est un document médico-légal descriptif dont finalité est de prouver l´existence du dommage et de permettre à la victime d´obtenir ce que de droit. L´objectif de notre étude était d'étudier le contenu et d'évaluer la qualité de rédaction des certificats médicaux initiaux. Il s´agissait d´une étude rétrospective et descriptive sur une période de 18 mois allant de janvier 2015 à juin 2016. Nous avons colligé 450 certificats médicaux initiaux parvenus au service de médecine légale du centre hospitalier universitaire de Charles Nicolle à Tunis. La qualité des certificats médicaux initiaux a été évaluée grâce à un canevas qui a permis de leur attribuer une note sur 30. Les certificats médicaux initiaux évalués étaient majoritairement moyens. Les scores obtenus à partir du canevas que nous avons élaboré oscillaient entre 9,5 et 27,5/30 avec une moyenne de 18,59/30. Les médecins généralistes, les médecins ayant une spécialité d´organe comme les ophtalmologistes ou encore les neurochirurgiens et les médecins qui ont utilisé le support du ministère de la santé ont rédigé de meilleurs certificats. De même nous avons noté une qualité de rédaction meilleure lorsqu´il s´agit de coups et blessures volontaires. Notre étude a montré que la majorité des certificats médicaux initiaux ne sont pas conformes aux recommandations de rédaction. Ces insuffisances sont probablement liées au fait que les médecins n´ont jamais bénéficié d´une formation médico-légale adéquate.

## Abstract (en)

Initial medical certificate is a descriptive medical and legal document whose purpose is to prove the existence of a damage and to enable the victim to access his or her right. The purpose of our study was to study the content and to evaluate the writing quality of initial medical certificates. We conducted a retrospective and descriptive study over an 18-month period, from January 2017 to June 2018. We collected data from 450 initial medical certificates at the Forensic Medicine Department of the Charles Nicolle University Hospital in Tunis. The quality of the initial medical certificates was assessed using a template that allowed to assign them a score out of 30. A mean score was mainly assigned to initial medical certificates Template scores ranged between 9.5 and 27.5/30 with an mean of 18.59/30. General practitioners and specialist physicians, such as ophthalmologists, neurosurgeons and doctors using the support provided by the Ministry of Health wrote better certificates. Similarly, we noted better quality of writing when certificates dealt with intentional assaults and injuries. Our study shows that the majority of initial medical certificates does not conform to editorial guidelines. These shortcomings are probably related to the fact that doctors have never received adequate medical-legal training.

## Introduction

Le certificat médical initial (CMI) est le premier document qui contient des informations relatives à l´état de santé d´une personne victime de violence, d´un accident de la circulation ou d´un accident de travail [1]. La finalité du CMI est de prouver l'existence du dommage et de permettre à la victime d´obtenir ce que de droit. Tout médecin peut être amené à rédiger des CMI. Il s´agit d´un acte médical qui engage la responsabilité pénale, civile et ordinale du médecin rédacteur [2]. La plupart des médecins trouvent la rédaction des CMI laborieuse, plus particulièrement lors de la détermination de la durée de l´Incapacité Temporaire Totale (ITT) [3]. Ces difficultés s´expliquent, en partie, par le fait qu´il n´existe aucun barème de référence qui permettrait de déterminer la durée de l´ITT avec exactitude [4]. Notre étude a eu pour but d'étudier le contenu et d´évaluer la qualité de rédaction des CMI parvenus au service de médecine légale du centre hospitalier universitaire (CHU) Charles Nicolle de Tunis.

## Méthodes

**Type d´étude**: il s´agit d´une étude rétrospective et descriptive qui s´étend sur une période de 18 mois allant du mois du premier janvier 2015 au 30 juin 2016. Nous avons colligé les CMI rédigés par des médecins de spécialités différentes au sein de structures sanitaires différentes et exerçant dans les neuf gouvernorats du nord de la Tunisie et qui sont parvenus au service de médecine légale du CHU Charles Nicolle de Tunis dans le cadre d´expertises pour évaluation du dommage corporel. L´étude a inclus les CMI qui sont parvenus au service de médecine légale du CHU Charles Nicolle de Tunis durant la période d´étude et qui ont été délivrés suite à des actes de violence ou des accidents de la circulation. L´étude a exclu les CMI délivrés suite à un accident domestique, un accident de travail, une maladie professionnelle ou une agression sexuelle.

**Analyse du certificat médical initial**: les données ont été recueillies à partir d´une fiche signalétique (canevas) conçue pour cette étude (Annex 1) permettant d´évaluer la qualité des CMI en attribuant un score sur 30. La qualité du CMI est «excellente» si le score est compris dans l´intervalle ]25-30]; «bonne» si le score est compris dans l´intervalle ]20-25]; «moyenne» si le score est compris dans l´intervalle ]15-20]; «médiocre» si le score est inférieur ou égal à 15. Nous avons noté des éléments pouvant influer sur la qualité du CMI et expliquer les disparités: l´âge et le sexe de la victime, la qualité du médecin rédacteur (sa spécialité), le type de la structure sanitaire et le type du traumatisme (accident de la circulation ou violence).

**L´analyse statistique**: l´analyse statistique a été effectuée avec le logiciel statistique Statistical Package for Social Sciences (SPSS) for Windows version 18.0. Le test du khi-deux a été employé pour comparer des proportions. Les tests ont été considérés comme significatifs quand la valeur de p était inférieure à 0,05.

## Résultats

Un total de 450 CMI a été colligé. Les accidents de la circulation constituaient 85,3% des motifs de rédaction, les violences volontaires 14,7%.

**La répartition des médecins signataires**: les médecins rédacteurs étaient représentés majoritairement par les orthopédistes (39,8%) et les médecins généralistes (28,2%). D'autres spécialités étaient aussi concernées tels la chirurgie (7,3%), la médecine d´urgences (6,9%), l´oto-rhino-laryngologie (4,7%), la chirurgie pédiatrique (2,9%), la chirurgie maxillo-faciale (2,7%), la réanimation (2,2%), l´ophtalmologie (1,8%) el la neurochirurgie (1,1%).

**La qualité de la rédaction**: les scores attribués aux CMI ont varié de 9,5 à 27,5 avec une moyenne générale de 18,59 (Tableau 1). La moyenne des scores de CMI rédigés par les orthopédistes était inférieure à la moyenne générale (17,30<18,59); celle des médecins généralistes était légèrement supérieure à la moyenne générale (19,22>18,59). Nous avons comparé la moyenne des scores des médecins généralistes, des médecins ayant une spécialité polyvalente et des médecins ayant une spécialité d´organe (Tableau 2). Les rédacteurs de CMI ayant une spécialité d´organe ont obtenu une moyenne supérieure à la moyenne générale (19,45>18,59) et à la moyenne des médecins ayant une spécialité polyvalente (18,06). Le test de Khi -deux a montré que les scores dépendent de façon statistiquement significative (p<0,05) de la nature des spécialités des médecins rédacteurs. Les mesures d'associations (Phi et V de Cramer) confirment les tests du Khi-deux: la relation entre le score et la variable « spécialité » est statistiquement significative (p < 0,05). Les CMI colligés on été rédigés dans des CHU dans 86,2% des cas, 8,2% des CMI ont émané d´hôpitaux régionaux et 3,1% de cabinets privés. Les moyennes des certificats rédigés dans les hôpitaux régionaux et de circonscription étaient plus élevées que les autres centres de soin. Le test de Khi-deux révèle néanmoins que les scores ne sont pas significativement liés au centre de soin (p>0,05) (Tableau 2). Moins de la moitié (44,9%) des médecins ont utilisé un papier-en-tête du Ministère de la Santé prévu à cet effet. Les autres, soit 55,1%, se sont servis de modèles qu´ils ont élaboré eux-mêmes ou d´autres supports inadaptés. La moyenne des scores des certificats rédigés sur le papier en tête du système de santé tunisien était plus élevée que lors de l´usage d´autres supports (19,9>17,5) (Tableau 2). Le test du khi deux a montré que la moyenne était statistiquement liée au support utilisé (p<0,05).

**Tableau 1:** répartition des scores obtenus

Scores obtenus	Effectifs	Appréciation
[25-30]	4(0,89%)	Excellent
[20-25]	107(23,8%)	Bon
[15-20]	305(67,8%)	Moyen
<ou =15	34 (7,6%)	Médiocre

**Tableau 2:** score en fonction des variables

Variables	Effectifs	Moyenne des scores obtenus	Test de Khi-deux
**Spécialité du médecin**			
Médecine générale	127	19,22	P<0,05
Spécialités polyvalentes	268	18,06	
Spécialités d´organe	55	19,45	
**Type du traumatisme**			
Accident	384	18,42	P<0,05
Violence volontaire	66	19,57	
**Support utilisé: Formulaire du Ministère de la Santé**			
Non	248	17,45	P<0,05
Oui	202	19,87	
**Structure sanitaire**			
CHU	388	18,43	P=0,486
Hôpital régional	37	19,93	
Hôpital de circonscription	3	21,83	
Centre de santé de base	6	19,92	
Clinique privée	2	17,75	
Cabinet privé	14	18,25	

### Les erreurs de rédaction

**Le volet administratif**: le motif de rédaction a été précisé dans 38,2% des CMI (à la demande de la victime ou sur réquisition judiciaire). L´écriture a été jugée illisible ou difficilement déchiffrable dans 22,4%des cas. La moitié (50,7%) des médecins signataires ont utilisé des abréviations non conventionnelles. Le nom du praticien a été indiqué dans 99,6% des cas, sa qualité dans 91,3% des cas. Un seul CMI a été rédigé par un médecin non doctorant (interne). Concernant l´identité de la victime, 26,9% des médecins signataires ont noté le numéro de la carte d´identité nationale (CIN) de la victime, celui du tuteur légal si celle-ci est mineure ou encore la mention « déclare se nommer » en l´absence de papiers d´identité.

**Le volet médical**: la date du traumatisme a été mentionnée dans 86,4% des cas. La date de l´examen a été précisée dans 75,8% des CMI. La date de rédaction a été indiquée dans 92,9% des CMI. Le délai de constatation des lésions n´a pu être déterminé que si les dates du traumatisme et de l´examen ont toutes les deux été mentionnées, soit dans 63,5% des certificats. La majorité (96,1%) des CMI étaient rédigés entre 0 et 3 jours après le traumatisme. Le CMI était rédigé après 3 à 7 jours dans 2,4% des cas, et dans des délais supérieurs à 7 jours dans 1,4% des cas. Le type de traumatisme (accident de la circulation ou violence volontaire) a été précisé dans 97,6% des cas. Les faits ont été rapportés au conditionnel ou au déclaratif dans 94,6% des cas, l´indicatif a été employé dans 5,4% des cas. Des termes manquant d´objectivité ou à connotation judiciaire étaient utilisés dans 22,7% des cas, tel que l´utilisation de l´expression « victime sauvagement tabassée ». La nature des lésions a été précisée dans 84,7% des cas, leurs localisations par rapport aux repères anatomiques dans 88,4% des cas. Les éléments permettant de dater les lésions comme la coloration des ecchymoses n´ont été mentionnés que dans 1,6% des cas. Les dimensions des lésions ont été indiquées dans 12,7% des CMI colligés. Les formes de celles-ci avaient été décrites dans 5,8% des cas. La localisation des lésions par rapport aux repères anatomiques et leur latéralité ont été précisées dans respectivement 88,4% et 86,7% des cas. Les rubriques non mentionnées dans le modèle du Ministère de la Santé tels que les doléances des consultants, leurs antécédents personnels et les traitements administrés ont été rapportées dans respectivement 11,3%, 6% et 16,9% des cas. La durée de l´ITT évaluée a été précisée dans 99,3% des cas. Le libellé « sous réserve de complications » a été employé dans 98,9% des certificats. L´évaluation de la durée d´ITT a été considérée comme inadaptée aux lésions décrites dans 2,9% des cas.

## Discussion

Le CMI est un certificat médical descriptif qui doit être rédigé le plus rapidement possible après l´épisode traumatique. Plus ce délai est long, plus le lien de causalité entre les lésions observées et les faits rapportés est incertain [5]. Lorsque le certificat est demandé sur réquisition par une autorité judiciaire ou administrative, le médecin requis doit s´en tenir aux questions de la mission, informer la personne concernée de cette mission et obtenir son consentement. Le certificat doit comporter: l´identité et la fonction de l´autorité requérante, l´énoncé précis de la mission, la signature du requérant, la date et le sceau. Le CMI peut être rédigé au décours de blessures volontaires (coups et blessures) ou involontaires (les accidents de circulation et les accidents du travail). Les violences conjugales sont une entité particulière et constitue un facteur aggravant sur le plan pénal. En effet, l´article 218 du code pénal [6] stipule que si l´auteur de l´agression est son conjoint la peine sera doublée. La circulaire 2014/39, publié par le Ministère de la Santé le 30 Mai 2014, avait prévu un modèle destiné aux CMI rédigés à la suite de violences conjugales et comporte la mention *«elle déclare avoir été agressé par son mari»*. Ce type de formulaire pourrait servir d´aide-mémoire pour le médecin dans la rédaction du certificat, lequel doit rester objectif et se limiter à une description détaillée des lésions constatées.

**La répartition des médecins signataires**: l´étude de la répartition des rédacteurs dans notre étude a montré que les médecins les plus sollicités pour la rédaction des CMI sont les orthopédistes (39,8% des médecins) et dans une moindre mesure les médecins généralistes (28,2%). Cette répartition peut être expliquée par le fait que les accidents de la circulation et les actes de violences provoquent souvent des traumatismes ostéo-articulaires qui sont pris en charge par des orthopédistes. En France, la majorité des CMI sont rédigés par des médecins généralistes; ceci s´explique par le fait qu´ils sont souvent recrutés dans les unités médico-judiciaires (UMJ) [7].

**La qualité de la rédaction**: notre étude a montré que les médecins généralistes avaient une moyenne de score supérieure à la moyenne générale et rédigent donc des CMI plus complets et plus conformes aux recommandations que les médecins spécialistes. Les orthopédistes avaient obtenu une moyenne de scores relativement basse. Ces résultats concordent avec les données de la littérature [8,9], dont une étude française réalisée en 2006 qui a montré que les médecins généralistes étaient majoritairement conscients des implications juridiques des CMI. Selon cette étude, 94% d´entre eux connaissaient les conséquences juridiques pénales de la durée de l'incapacité temporaire de travail. L´acquisition de ces connaissances est facilitée par le suivi de formations spécifiques concernant la rédaction de CMI. Ces formations sont souvent dispensées par des médecins légistes et visent non seulement à enseigner les règles de bonne rédaction mais aussi à sensibiliser ces médecins aux conséquences juridiques de tels certificats. Les médecins ayant une spécialité d´organe (ophtalmologie, oto-rhino-laryngologie (ORL), neurochirurgie, chirurgie cardio-vasculaire, chirurgie maxillo-faciale) ont obtenu une meilleure moyenne de scores que les généralistes et les médecins ayant une spécialité polyvalente. Ceci peut être expliqué par le fait que l´examen clinique est ciblé à un organe ce qui leur permet d´être plus précis dans leur description et que les examens complémentaires spécialisés tels que le scanner cérébral pour les neurochirurgiens ou encore le fond d´œil pour les ophtalmologistes sont pratiquement toujours indiqués et retranscrits dans les certificats.

La plupart des CMI colligés (86,2%) ont été rédigés dans des CHU. Ces CMI sont rédigés aux urgences ou aux consultations des différents services des CHU. En France, suite à la réforme de la médecine légale en décembre 2010 [10], la majorité des CMI sont rédigés au sein des UMJ dédiées à la médecine légale du vivant et dont l´activité principale consiste à répondre aux réquisitions judiciaires. Notre étude n´a pas montré de liens tangibles entre la qualité rédactionnelle des CMI et la structure sanitaire où sont affectés les médecins rédacteurs. D´autres études faites en France ont montré que les CMI qui ont été rédigés au sein de structures spécialisées (UMJ) étaient de meilleure qualité [1]. La moyenne des scores obtenue dans les cas de violences volontaires était meilleure que celle obtenue au décours de blessures involontaires. Une plus grande vigilance semble être apportée lorsque le consultant est victime d´un acte de violence dont les conséquences sont plus lourdes autant pour la victime qui veut faire valoir ses droits que pour l´auteur des violences qui encourt des sanctions graves. Notre étude a montré que la qualité rédactionnelle des CMI dépendait, entre autre, du support utilisé. L´existence de rubriques qu´il suffit de compléter dans le support fourni par le Ministère de la Santé [11] permet au médecin rédacteur de ne pas occulter les données nécessaires. Plusieurs données manquent toutefois sur le support du Ministère de la Santé, tels que le numéro d´inscription au Tableau de l´Ordre des Médecins, les antécédents, les doléances du patient, les avis spécialisés, les examens complémentaires ou le traitement prescrit. Nous avons retrouvé les mêmes résultats dans une autre étude française [12], qui a également conclu que l´utilisation d´un modèle préétabli reprenant les rubriques nécessaires à l´utilisation rationnelle des données recueillies améliore considérablement la qualité des CMI.

### Les erreurs de rédaction

**Le volet administratif**: nous avons considéré que 22,4% des CMI étaient illisibles, c'est-à-dire que l´écriture était difficile à lire ou réellement indéchiffrable. La dactylographie pourrait constituer une bonne alternative pour rendre leur lecture plus facile et éviter les erreurs de déchiffrage. Dans les études françaises, tous les certificats écrits à la main ont été considérés comme illisibles, ce qui correspond à 75% des CMI dans l´étude Doriat *et al*. [1] en 2003 et à la totalité des CMI dans l´étude Guérant *et al*. [7] en 2016. Plus de la moitié des CMI colligés (50,7%) contenaient des abréviations. Si ces abréviations peuvent sembler évidentes pour les membres du corps médical, elles le sont beaucoup moins pour les autorités judiciaires qui sont aussi amenés à lire ces CMI et peuvent être faussement interprétées. Le nom du praticien a été indiqué dans 99,6% des cas et sa qualité dans 91,3% des cas. L´étude Ben Taïeb *et al*. [13] a trouvé que le nom du médecin figurait dans 99,6% des certificats et leurs grades dans 81,6% des cas. L´étude de Blaise *et al*. [14] a montré que seulement 35,3% des médecins ont indiqué leurs noms (ils se sont contentés du cachet). Une minorité de médecins (26,9%) ont indiqué le numéro de la carte d´identité nationale (CIN) de la victime ou ont utilisé une formule du type « déclare se nommer » à défaut de pièce d´identité. L´étude Ben Taïeb *et al*. [13] a montré que 24,8% des CMI étudiés comportaient le numéro d´une pièce d´identité contre 74% des cas dans l´étude Blaise *et al*. [14]. L´absence de numéro de CIN est une insuffisance grave sur le plan pénal et peut, le cas échéant, causer l´invalidité judiciaire du certificat. La conformité des critères administratifs aux recommandations dans différentes études figure dans le Tableau 3.

**Tableau 3:** conformité des critères administratifs, des examens cliniques et des rubriques non mentionnées dans le support du Ministère de la Santé aux recommandations dans différentes études

Critères	Etude Ben Taīeb *et al*. [13]	Etude Blaise *et al*. [14]	Etude Ben Lassoued *et al*.[15]	Etude Guérant *et al*. [7]	Notre étude
**Conformité des critères administratifs**					
Utilisation du support du Ministère de la Santé	82,6%	-	-	-	44,9%
Nom du médecin rédacteur	99,6%	35,7%	-	-	99,6%
Qualité du médecin	81,6%	99,72%	-	-	91,3%
Présence d´abréviations	47,5%	-	-	-	50,7%
Numéro CIN du consultant	24,8%	74%	-	-	26,9%
Age du consultant	18%	100%	-	-	55,4%
Circonstances de rédaction	32%	100%	-	-	38,2%
Termes subjectifs	24,8%	0%	-	-	22,7%
**Conformité des examens cliniques**					
Nature de la lésion	-	-	-	100	84,7
Siège des lésions	92,6	-	57,6	99,8	88,4
Couleur/Datation	3,9	-	0	86,4	1,6
Dimensions	26	-	39,8	89,4	12,7
Forme de la lésion	25,3	-	42,1	64,4	5,8
**Conformité des rubriques non mentionnées dans le support du Ministère de la Santé**					
Antécédents	1,2	-	-	-	6
Doléances	16,2	98,9	1,7	-	11,3
Avis spécialisés	0	11 ,1	12,5	-	9,1
Examens complémentaires	63,2	32,5	62,5	-	33,6
Traitement	17,7	-	-	-	16,9

Le volet médical: la date de l´examen est la date la moins fréquemment précisée (75,8% des cas). L´étude Ben Taïeb *et al*. [13] a montré la même tendance: la date de l´examen a été mentionnée dans 80,4% des cas. Ceci peut être expliqué par le fait que les médecins estiment qu´il est évident que le certificat soit rédigé le même jour que l´examen alors que ce n´est pas toujours le cas. Les médecins ont tous précisé le type de traumatisme. Il s´agissait d´un accident de la circulation dans 85,3% des cas et d´un acte de violence dans 14,7% des cas. L´étude Ben Taïeb [13] a trouvé des chiffres comparables: 75,6% d´AVP et 21% de violences. Des termes à connotation subjective ou judiciaire ont été utilisés dans 22,7% des CMI. L´étude Blaise *et al*. [14] a trouvé que 95,73% des médecins ont utilisé le conditionnel et que seulement 1,42% des CMI contenaient des termes à connotation judiciaire. Le médecin doit rester objectif et impartial et ne doit pas impliquer formellement et nommément des tiers en cause, ni utiliser des termes à connotation judiciaire ou mentionner des éléments inutiles ou sans conséquences médico-légales. La détermination de la responsabilité de l´auteur des faits présumés doit rester de la compétence du magistrat et non du médecin.

Concernant l´examen clinique, la nature exacte des lésions, leurs localisations par rapport aux repères anatomiques, leurs dimensions, leurs formes et leurs colorations sont des éléments inconstamment retrouvés dans les CMI colligés dans notre étude. Pourtant, ces informations permettent de dater les blessures et de s´assurer de l´imputabilité du traumatisme, orientent vers l´objet vulnérant et nous permet ainsi de vérifier la concordance des lésions avec les faits rapportés par la victime. Ces précisions permettent aussi d´évaluer la gravité de la lésion et influe donc sur la durée de l´ITT. Nous avons comparé la conformité de l´examen clinique observée dans notre étude avec l´étude Ben Lassoued [15] où les CMI ont été colligés aux urgences de l´hôpital militaire de Tunis, l´étude Ben Taïeb [13] réalisée à Monastir et une étude française (Guérant *et al*.) où les CMI ont été rédigés dans des structures sanitaires spécialisées (UMJ) (Tableau 3). On remarque que les examens cliniques des CMI qui ont été colligés dans les UMJ (étude Guérant *et al*.) sont nettement plus conformes aux recommandations que les CMI qui ont été rédigés dans d´autres services [13,15].

Les rubriques non mentionnées dans le support du Ministère de la Santé (numéro d´inscription au Tableau de l´Ordre des Médecins, antécédents médico-chirurgicaux, doléances, examens complémentaires, recours à des avis spécialisés, traitements) sont des éléments essentiels de l´expertise de dommage corporel. Nous avons comparé, dans le tableau ci-dessous, la conformité des rubriques non mentionnées dans le formulaire du Ministère de la Santé dans différentes études afin de confirmer ou d´infirmer le rôle du support dans la négligence de ces critères. On remarque que, mis à part les doléances qui sont considérablement plus souvent rapportées dans l´étude française (étude Blaise *et al*. [14]), ces rubriques semblent généralement négligées indépendamment du papier en tête utilisé (Tableau 3).

**Les implications juridiques des CMI**: l´ITT correspond à la période, en jours, pendant laquelle une victime d´agression ou d´accident ne peut réaliser au moins une des activités nécessaires à sa vie quotidienne, comme se déplacer, se nourrir, se laver, s´habiller et aller aux toilettes. L´ITT traduit de façon quantitative le retentissement fonctionnel des blessures détaillées par le médecin. Elle ne reflète pas la violence infligée mais ses conséquences [16]. La durée de l´ITT repose uniquement sur les données de l´examen clinique. Elle est indépendante des circonstances de l´événement traumatique (accident, agression…). Il n´existe actuellement aucune échelle d´évaluation objective de l´ITT à la disposition des médecins lors de la rédaction de CMI de coups et blessures. Ceci pourrait expliquer la grande disparité entre les ITT attribuées par les médecins pour les mêmes lésions [17]. Dans le cadre d´une procédure judiciaire, l´ITT permet au magistrat d´apprécier la gravité des violences subies, de qualifier l´infraction (contravention ou délit) et donc de déterminer la juridiction de jugement compétente et les peines encourues par l´auteur des faits de violences. En Tunisie, aucune barrière en jours n'existe, et c'est le magistrat qui qualifie les violences de légères ou graves.

## Conclusion

Notre étude a montré que la majorité des CMI ne sont pas conformes aux recommandations de rédaction. Ces insuffisances sont probablement liées au fait que les médecins n´ont jamais bénéficié d´une formation médico-légale adéquate. L'effort de formation doit alors être accentué et l'enseignement de la rédaction du CMI doit être intégré dans le cadre des études médicales. Des formations en rédaction de CMI au cours de séminaires doivent être multipliées en privilégiant toujours des exercices ludiques et interactifs et en évaluant la progression à l´aide d´un post-test. Il serait aussi utile d´envisager l´élaboration d´un barème indicatif pour la détermination de la durée de ITT afin d´homogénéiser les durées attribuées par les médecins rédacteurs.

### Etat des connaissances sur le sujet

- *Le certificat médical initial est un document médico-légal descriptif;*
- *Tout médecin, généraliste ou spécialiste doit être capable de rédiger un CMI dans les règles de forme et de fond;*
- *La finalité du certificat médical initial est de prouver l'existence du dommage et de permettre à la victime d´obtenir ce que de droit*.

### Contribution de notre étude à la connaissance

- *Notre étude a montré que la majorité des certificats médicaux initiaux ne sont pas conformes aux recommandations de rédaction, les certificats évalués étant majoritairement moyens;*
- *Les médecins généralistes, les médecins ayant une spécialité d´organe (ophtalmologistes, neurochirurgiens, etc) ont rédigé de meilleurs certificats;*
- *La qualité de rédaction était meilleure lorsqu'il s'agit de coups et blessures volontaires*.

## Annex

**Annex 1**: canevas d´évaluation des CMI
